# Alcohol Use Disorder and the Gut–Brain Axis: A Narrative Review of the Role of Gut Microbiota and Implications for Treatment

**DOI:** 10.3390/microorganisms13010067

**Published:** 2025-01-02

**Authors:** Shikha Shukla, Cynthia L. Hsu

**Affiliations:** 1 Department of Medicine, University of California San Diego, La Jolla, CA 92093, USA; 2 Department of Medicine, VA San Diego Healthcare System, San Diego, CA 92161, USA

**Keywords:** short-chain fatty acids, tryptophan metabolism, neurotransmitters, toll-like receptors, neuroinflammation, glial cells

## Abstract

Alcohol use disorder (AUD) affects millions of people worldwide and can lead to deleterious physical and social consequences. Recent research has highlighted not only the effect of alcohol on the gut microbiome, but also the role of the gut microbiome and the gut–brain axis in the development and maintenance of alcohol use disorder. This review provides an overview of the reciprocal relationship between alcohol consumption and the gut microbiome, including the effects of alcohol on gut microbial composition, changes in gut microbial metabolites in response to alcohol consumption, and how gut microbial metabolites may modulate alcohol use behavior. We also discuss the gut-mediated mechanisms of neuroinflammation that contribute to and result from AUD, including disruption of the intestinal barrier, toll-like receptor signaling, and the activation of glial cells and immune cells. Finally, we review the current evidence on gut microbial-directed therapies for AUD and discuss the implications of this research for our understanding of the pathophysiology of AUD and future research directions.

## 1. Introduction

Alcohol use disorder (AUD) is a major global health concern characterized by persistent and excessive alcohol consumption, often marked by a loss of control, including the development of tolerance and intense cravings [1,2,3]. AUD is often comorbid with other physical and mental health issues [4] and the consequences of AUD include significant impairment in daily functioning, strained relationships, and reduced productivity. The etiology of AUD is multifactorial, involving genetic, prenatal, and environmental factors, as well as comorbid mental health conditions and stress. Emerging research implicates the gut microbiome as an additional key risk factor for AUD.

The gut–brain axis describes the complex, bidirectional communication network between the central nervous system and the gastrointestinal tract with its trillions of microbial inhabitants. Substances that are consumed orally, including food and alcohol, have a profound impact on the composition and function of the gut microbiota, which in turn can modulate brain activity and behavior, potentially perpetuating dietary habits [5,6]. For example, in response to food consumption, the gut microbiota can modulate the secretion of appetite-related hormones, such as glucagon-like peptide-1, peptide YY, and cholecystokinin, which, in turn, act on the central nervous system to increase satiety and decrease food intake [7,8,9,10]. This reciprocal relationship is also true in chronic heavy alcohol consumption and AUD.

Chronic alcohol consumption in individuals with and without alcoholic liver disease can disrupt the balance of gut microbiota, promoting the growth of aerobic and anaerobic bacteria and altering the composition of mucosa-associated microbiota. This disruption leads to changes in microbial metabolites, such as short-chain fatty acids, indoles, and tryptophan, which in turn influence the production and activity of neurotransmitters. These changes play a critical role in regulating mood, cognition, and addiction, and may contribute to both cognitive impairments and addictive behaviors [11,12,13]. These disruptions can trigger neuroinflammation through multiple mechanisms, including increased gut permeability, microglial activation, and astrocyte dysfunction [14,15]. Ultimately, these changes may contribute to the sustained pattern of alcohol consumption.

**Figure 1:**
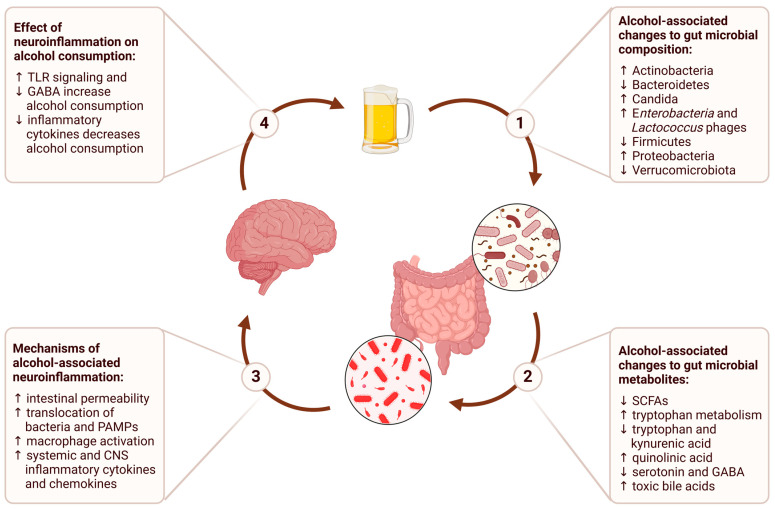
The gut–brain axis in alcohol use disorder describes a bidirectional communication network where alcohol consumption alters gut microbial composition, leading to changes in gut microbial metabolites that contribute to alcohol-associated neuroinflammation, and ultimately perpetuate a cycle of persistent alcohol consumption.

In this review, we will examine each stage of this process, from the impact of alcohol consumption on the gut microbiome to the downstream effects on brain function and behavior (Figure 1). We will conclude with a discussion of the therapeutic implications of the gut–brain axis and potential future directions for research on alcohol use disorder.

## 2. Gut Microbial Composition in Alcohol Use Disorder

The gastrointestinal tract is home to a vast array of microorganisms, including bacteria, archaea, fungi, and viruses, collectively forming the gut microbiota [16,17]. These microbial communities are crucial for synthesizing essential vitamins and amino acids and facilitating the breakdown of macromolecules, while also playing key roles in energy production, drug and toxin metabolism, and the maintenance of the intestinal barrier [18,19,20,21]. Although there is a significant role of diet, lifestyle, and genetic predispositions on the gut microbiome of an individual, both acute and chronic alcohol consumption, even in the absence of liver disease, can profoundly disrupt composition and diversity of the bacterial [22,23], fungal [24], and viral microbiomes [25,26].

Early investigations documented that the levels of Gram-negative anaerobic bacteria in jejunal samples from individuals with alcohol use disorders are markedly higher than in healthy controls [27]. Subsequent studies have confirmed that chronic ethanol consumption leads to decreased abundance of the Bacteroidetes and Firmicutes phyla, alongside increases in Gram-negative Proteobacteria and Gram-positive Actinobacteria [28]. Chronic alcohol consumption is also associated with alterations in the gut fungal microbiome, evidenced by the elevated relative abundances of *Candida*, *Debaryomyces*, *Pichia*, *Kluyveromyces*, and *Issatchenkia* in the gut microbiota of individuals with AUD compared to healthy controls [24]. Furthermore, following a period of abstinence, the gut microbiota of individuals with AUD exhibited reduced abundances of *Candida* and *Malassezia*, suggesting a reversible impact of alcohol consumption on the gut fungal community. Lastly, analysis of the fecal virome in individuals with AUD revealed a positive correlation between the abundance of *Enterobacteria* and *Lactococcus* phages and the severity of liver disease [26].

Alcohol-induced gut dysbiosis leads to intestinal barrier dysfunction, which allows for increased translocation of microbial components to the liver and can exacerbate the progression of liver disease [29,30]. In the presence of concurrent liver disease, continued alcohol consumption is associated with a reduction in beneficial commensal bacteria [31] and an increase in microbiota linked to endotoxin production, such as cytolysin-producing *Enterococcus faecalis* [32] and candidalysin-producing *Candida albicans* [33]. Both cytolysin and candidalysin can cause direct hepatocyte injury and death, and also trigger immune activation that exacerbates tissue damage. Alcohol consumption can significantly alter the composition of the gut microbiome, with implications for host health and disease susceptibility.

## 3. Gut Microbial Metabolites in Alcohol Use Disorder

Alcohol consumption not only affects gut microbial composition, but also microbial functions and metabolites. The gut microbiome produces a vast array of metabolites, including short-chain fatty acids, amino acid derivatives, neurotransmitters, bile acids, and other bioactive compounds, which can exert effects locally in the gastrointestinal tract or systemically through absorption in the bloodstream. For example, short-chain fatty acids produced by the gut microbiome can modulate the immune system, regulate inflammation, and even influence brain function and behavior. Other metabolites, such as tryptophan-derived compounds, can affect the production of neurotransmitters and hormones essential for regulating mood, appetite, sleep, and other physiological processes. Alcohol-induced modifications in gut microbial metabolites can result in hepatic toxicity, increased intestinal permeability, and augmented inflammatory responses, which all play a multifaceted role in shaping brain function. Here, we will highlight some important changes in gut microbial metabolites and their downstream effects secondary to alcohol consumption.

### 3.1. Short-Chain Fatty Acids (SCFAs)

SCFAs, primarily acetate, propionate, and butyrate in the human gut, are key microbial metabolites produced by gut microbiota through the fermentation of dietary fiber. SCFAs are critical for enterocyte health and maintenance of the intestinal barrier. In both humans with alcohol use disorder (AUD) and animal models of AUD, scientists have found significantly reduced levels of fecal SCFAs in individuals with AUD, as well as a decrease in SCFA-producing bacteria alongside an increase in pro-inflammatory microbes [34,35]. SCFAs can regulate systemic inflammation directly through the production of various inflammatory mediators, including cytokines, chemokines, and eicosanoids, through their action on G protein-coupled receptors (GPCRs) such as FFAR2, FFAR3, GPR109, and Olfr78 [36,37]. SCFAs also indirectly regulate systemic inflammation through maintenance of the intestinal barrier—a reduction in fecal SCFAs leads to increased intestinal permeability, which can heighten the risk of liver injury and systemic inflammation [29]. Increased gut permeability and systemic inflammation, in turn, are positively correlated with symptoms such as depression and alcohol craving, which may contribute to the development and maintenance of alcohol dependence [38].

In addition to affecting gut permeability, SCFAs may also impact alcohol consumption in AUD via several different mechanisms. For example, acetate metabolized from ethanol can be converted to acetyl-CoA and deposited into the histones of neurons [39]. In the hippocampus, alcohol-induced histone acetylation has been found to upregulate transcriptional programs important for alcohol-related associative learning [39]. Acetate may also have an effect on cerebral blood flow, which could modulate neuroinflammation [40,41,42]. Although the effects of acetate on the epigenetic modulation of neuronal genes and its involvement in driving neuroinflammation have been extensively studied in mouse models, one of the limitations of these findings is that they still require validation in human studies.

SCFAs can also travel through the blood–brain barrier (BBB) to directly affect neuronal health. The endothelial cells of the BBB contain monocarboxylate transporters (MCTs) that enhance BBB permeability to SCFAs. Butyrate has been shown to benefit mice exposed to chronic stress by effectively counteracting depressive behaviors associated with prolonged stress exposure [43]. Furthermore, administration of butyrate resulted in elevated serotonin levels in the hippocampus and an increase in the expression of brain-derived neurotrophic factor (BDNF) [44]. Both depression symptoms and serotonin levels are tightly interconnected with alcohol consumption in people with AUD.

SCFAs can affect behaviors related to stress and substance use, including addiction. In rats, sodium butyrate has been found to mitigate alcohol-induced liver damage and inflammation while also exhibiting antidepressant-like effects [45]. One specific SCFA, valeric acid, has been shown to decrease binge drinking and anxiety-like behaviors. Mice that were supplemented with valeric acid exhibited a 40% reduction in alcohol consumption and had blood alcohol levels that were 53% lower [46]. Additionally, a recent study found that decreased levels of fecal isovalerate, an isomer of valeric acid, were associated with depression in humans [47]. Although the precise mechanisms through which SCFAs influence alcohol consumption and addiction remain unclear, they may involve the modulation of gene expression and epigenetic alterations in the brain.

### 3.2. Tryptophan Metabolites

Tryptophan, an essential amino acid, is not produced by animal cells and is primarily obtained through dietary intake. The gut microbiota play a crucial role in tryptophan metabolism, converting it into bioactive compounds such as aryl hydrocarbon receptor (AhR) ligands and serotonin when the indole ring remains intact [48]. However, when the indole ring is oxidatively cleaved, tryptophan is redirected into the kynurenine pathway, reducing its availability for neurotransmitter and indole synthesis [49].

In mammals, the majority of tryptophan is catabolized through the kynurenine pathway by indoleamine 2,3-dioxygenase 1 (IDO1) into kynurenine and downstream products such as kynurenic acid and quinolinic acid [50]. Gut microbiota are a critical stimulator of IDO1 activity—animals without gut microbiota demonstrate reduced tryptophan metabolism through the kynurenine pathway, an effect that is reversed upon recolonization [51]. Kynurenic acid is known for its neuroprotective and anticonvulsant effects. It acts as a competitive antagonist of N-methyl-D-aspartate (NMDA) receptors and activates G protein-coupled receptor 35, modulating cyclic AMP production and inhibiting N-type Ca^2^⁺ channels in sympathetic neurons and astrocytes to suppress inflammatory pathways [52,53]. In contrast, quinolinic acid is an NMDA receptor agonist that promotes inflammation and oxidative stress through the generation of reactive oxygen species, depletion of endogenous antioxidants, and promotion of lipid peroxidation [54,55].

In the context of AUD, alcohol consumption and chronic inflammation also stimulate the production of IDO1, leading to increased tryptophan metabolism via the kynurenine pathway [56,57]. This results in depletion of tryptophan and neuroprotective kynurenic acid, and the accumulation of neurotoxic quinolinic acid. One study observed significant changes in kynurenine metabolites following a 3-week detoxification program, including elevations in kynurenine and reductions in 3-hydroxykynurenine and xanthurenic acid, alongside increased quinolinic acid levels [58,59]. Furthermore, research in rats has shown that the metabolites 3-hydroxykynurenine, kynurenic acid, and 3-hydroxyanthranilic acid inhibit alcohol dehydrogenase, leading to the accumulation of toxic metabolite acetaldehyde and increased aversion to alcohol [60]. These findings suggest that the gut microbiota’s regulation of tryptophan metabolism via the kynurenine pathway in the context of alcohol consumption may contribute to alcohol-seeking behaviors through multiple mechanisms, including brain inflammation and acetaldehyde accumulation.

### 3.3. Neurotransmitters

The production and modulation of neurotransmitters such as serotonin, glutamate, and γ-aminobutyric acid (GABA) by gut microbiota is also affected by alcohol consumption. Most of the body’s serotonin is produced by enterochromaffin cells in the gut, and the gut microbiota play a crucial role in regulating serotonin production [61]. Studies utilizing germ-free mice have demonstrated that the absence of gut microbiota results in significantly reduced serotonin production in the colon and decreased circulating serotonin levels [62]. The gut microbiota is thought to modulate serotonin production directly through the induction of tryptophan hydroxylase 1 (TpH1) expression, a rate-limiting enzyme in the serotonin biosynthetic pathway [63]. Additionally, the production of secondary bile acids by gut microbiota may also contribute to the stimulation of serotonin biosynthesis. As discussed above, induction of tryptophan catabolism via the kynurenine pathway by alcohol consumption decreases tryptophan availability for serotonin synthesis, and this is exacerbated by alcohol-induced gut dysbiosis. For example, decreased levels of serotonin and serotonin metabolites were found in the hippocampus and serum of conventional mice compared to germ-free mice [64]. This dysregulation triggers neuroinflammation via serotonin binding to serotonin (5-HT) receptors on microglia [65].

Gut microbes can also regulate levels of GABA by consuming it via the GABA shunt, a metabolic pathway that converts GABA to succinate, as a carbon and nitrogen source, or by producing it as a way to modulate intracellular pH [66]. Germ-free animals exhibit significantly reduced luminal and serum, but not cerebral, GABA levels [67]. Chronic alcohol consumption leads to gut dysbiosis, which has been shown to impair the conversion of glutamate to GABA by beneficial bacteria like *Lactobacillus* and *Bifidobacterium*, subsequently reducing GABA levels and increasing neuronal excitability [68]. Additionally, the systemic inflammation caused by alcohol-induced increases in gut permeability and translocation of lipopolysaccharide products from the outer membrane of Gram-negative bacteria (discussed in more detail later in this review) attenuates GABA synthesis and receptor activity in the spinal dorsal horn [69]. A clinical study by Kirsten et al. found that brain GABA levels are inversely correlated with the severity of alcohol-associated liver disease, highlighting the importance of GABA in maintaining neural balance [70]. These findings suggest that alterations in the gut microbiota by alcohol consumption lead to dysregulation of neurotransmitters and may exacerbate the neuropsychiatric consequences of alcohol abuse.

### 3.4. Bile Acids

Primary bile acids are synthesized by hepatocytes and released via bile ducts into the gastrointestinal tract where they are metabolized by gut microbiota into secondary bile acids. The process of bile acid metabolism begins with bile acid deconjugation, or hydrolysis of the amino acid moiety, via gut bacterial bile salt hydrolases, which are highly conserved across all major gut microbial phyla [71]. Dysbiosis caused by chronic alcohol consumption disrupts bile acid homeostasis and leads to a systemic increase in bile acid levels [72]. Specifically, alcohol consumption is associated with elevated levels of toxic bile acids, including chenodeoxycholic acid, deoxycholic acid, and lithocholic acid, in enterohepatic circulation, which contribute to the pathogenesis of alcohol-associated liver injury. A recent study suggested that bile acids can not only affect the development of liver disease, but modulation of intrahepatic bile flow, including through the use of bile acid transport inhibitors, could also affect drinking behavior through the alteration of serum acetaldehyde levels [73].

## 4. Gut-Mediated Mechanisms of Neuroinflammation in Alcohol Use Disorder

**Figure 2:**
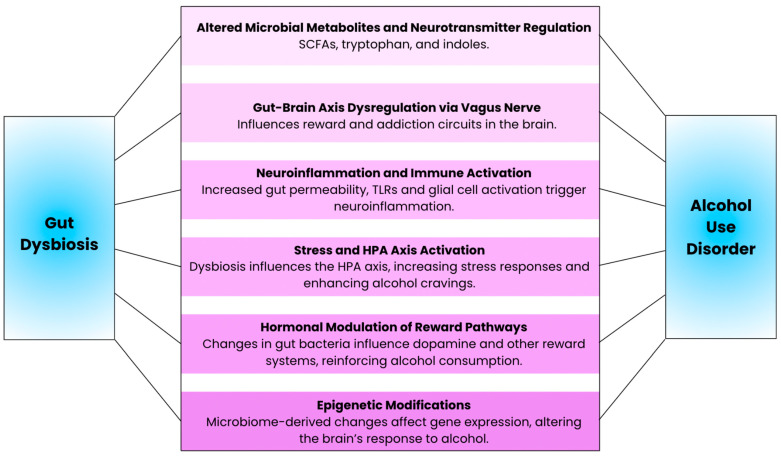
Illustration of the key mechanisms underlying the connection between gut dysbiosis and alcohol use disorder.

Modulation of gut microbial composition and metabolites by chronic alcohol consumption can lead to neuroinflammation through a variety of different mechanisms (Figure 2). First, alcohol-induced gut dysbiosis promotes systemic inflammation and, indirectly, neuroinflammation, by increasing intestinal permeability and allowing for the direct translocation of bacteria and inflammatory products, as well as pathogen-associated molecular patterns that trigger inflammatory signaling cascades. Second, alcohol consumption more directly causes neuroinflammation via damage to the blood–brain barrier and activation of microglia and astrocytes. In this subsection, we will review how alcohol consumption can induce neuroinflammatory responses that elicit adverse effects on neuronal health and cognitive behavior.

### 4.1. Disruption of the Intestinal Barrier

Several studies show that individuals with alcohol dependence exhibit increased gut permeability, which correlates with elevated levels of depression, anxiety, and cravings for alcohol [74,75]. Studies using animal models of alcohol-associated liver disease have demonstrated that modulating the gut microbiome with dietary fibers or probiotics such as *Lactobacillus GG* can reduce endotoxemia, inflammation, and liver damage by reducing gut permeability [76]. The intestinal barrier consists of tight junctions and adherens junctions between intestinal epithelial cells. Proteins such as claudin and occludin help maintain this barrier, allowing for the selective passage of nutrients while preventing the entry of harmful bacteria into the bloodstream. Alcohol consumption disrupts the intestinal barrier by altering the expression of tight junction-associated proteins such as ZO-1, claudin-1, claudin-5, and claudin-7 through the activation of PKCα [77,78]. Alcohol consumption also induces the production of reactive oxygen species and nitric oxide [79]. Additionally, alcohol upregulates CYP2E1 expression in gut epithelial cells and activates the nuclear factor kappa B (NF-κB) pathway, leading to cytoskeletal disruption and increased intestinal permeability [80]. As a result, harmful bacterial endotoxins, including lipopolysaccharides and peptidoglycans, are allowed to enter the systemic circulation, triggering systemic inflammatory responses [38,81].

Key cytokines, including tumor necrosis factor (TNF), interferon-gamma (IFN-γ), and interleukin-1β (IL-1β), act as pivotal mediators in this process. Chronic and acute ethanol exposure have been shown to increase TNF-α production, which can lead to glutamatergic excitotoxicity [82]. Additionally, IFN-γ enhances the effects of TNF-α by increasing the expression of TNF receptors on epithelial cells, amplifying intestinal permeability [83]. The upregulation of adhesion molecules such as ICAM-1, driven by IFN-γ, facilitates neutrophil migration into subepithelial spaces, exacerbating tissue damage and inflammation [84].

Alcohol also affects intestinal immune cells, which play an essential role in gut permeability. For example, alcohol induces M2b polarization of macrophages, which can contribute to inflammation and tissue damage [85]. Recent research highlights the importance of intestinal macrophages in regulating inflammation during dysbiosis and maintaining epithelial barrier function. These immune cells not only promote the survival of FOXP3+ regulatory T cells, which are essential for maintaining immune tolerance, but they also contribute to the preservation of epithelial barrier function [86].

In a study of patients with AUD, a subset of individuals exhibited increased gut permeability, and this was associated with altered gut microbiota composition and activity as well as higher scores of depression, anxiety, and alcohol craving after abstinence [74]. These results suggest the importance of gut barrier function in the development and maintenance of alcohol dependence.

### 4.2. Toll-like Receptors 

Toll-like receptors (TLRs) play a crucial role in the innate immune response, serving as key sensors for microbial detection and maintaining intestinal homeostasis. Among the various TLRs, TLR2, TLR3, TLR4, and TLR7 have been shown to be significantly affected by alcohol consumption [87]. Following ethanol intake, these receptors exhibit increased expression in the prefrontal cortex, a region vital for cognitive function, highlighting the impact of alcohol on neuroimmune signaling [88,89].

Concomitantly, these TLRs are also involved in alcohol addiction and AUD. Alcohol-induced activation of TLR3 has been shown to trigger a pro-inflammatory response in the hippocampus, characterized by elevated levels of cytokines such as IL-1β, IL-6, and TNF-α, which contribute to the development of neuroinflammation. Conversely, activation of TLR3 enhances voluntary alcohol consumption, suggesting a feedback mechanism that may contribute to continued alcohol consumption [90].

Acute activation of TLR7 has been shown to reduce ethanol intake and preference, likely due to an acute sickness response, whereas chronic activation of TLR7 via treatment with an agonist leads to tolerance and increased ethanol consumption [91]. Postmortem analysis of human brains from individuals with AUD has revealed elevated TLR7 expression and increased microglial activation, supporting the connection between alcohol, TLR signaling, and neuroinflammation [92].

Many studies have shown that inhibition of TLR4 decreases binge drinking behaviors [93,94]. For example, mice treated with lipopolysaccharide, a bacterial endotoxin, demonstrated persistent increases in alcohol consumption, whereas mice lacking CD14, a key component of TLR4 signaling, showed no increases in alcohol intake after treatment with lipopolysaccharide [95]. Additionally, rodents lacking TLR2 and TLR4 show protection against ethanol-induced neuroinflammatory responses and cognitive impairments, underscoring the importance of these receptors in mediating alcohol’s detrimental effects on the central nervous system [96,97].

### 4.3. Glial Cells and Neuroimmune Function

Glial cells, which include astrocytes and microglia, are non-neuronal cells that play a crucial role in maintaining the health and function of the nervous system by providing structural and metabolic support to neurons, regulating neurotransmitter activity, removing waste products, maintaining the blood–brain barrier, and contributing to the neuroimmune response. Many studies have highlighted their role in the gut–brain axis in the pathophysiology of AUD.

Astrocytes play a crucial role in regulating various aspects of AUD, including arousal, cortical sensory processing, and the experience of reward [98]. Studies in both mice and humans have shown that the ethanol-dependent brain exhibits significant dysregulation of genes associated with astrocyte function [99,100,101]. One such gene, glial fibrillary acidic protein (GFAP), an intermediate filament-III protein involved in astroglial cell activation following CNS injury and neurodegeneration, exhibits elevated expression in response to both acute and chronic ethanol exposure [102]. The expression of other genes, such as toll-like receptors and receptors for inflammatory cytokines, are also upregulated in astrocytes in response to alcohol. TNF-α and IL-1β disrupt astrocytic glutamate uptake, resulting in an accumulation of extracellular glutamate and the induction of neurotoxicity. The development and function of astrocytes are influenced by gut microbiota. The administration of lactic acid bacteria during the early developmental stages of rats significantly influences astrocyte maturation [103]. Moreover, changes in fecal microbiota and metabolites can affect astrocyte phenotypes and the development of age-related cognitive decline in mice [104].

The host microbiota also play a crucial role in maintaining microglial homeostasis. For example, germ-free mice exhibit widespread abnormalities in their microglia, including altered cell subtypes and an immature phenotype, which impairs innate immune responses [105,106]. Moreover, temporary removal of the host microbiota significantly alters microglial properties, and a limited diversity of microbiota leads to dysfunctional microglia [107]. The gut microbiome also influences the reciprocal transformation of microglial subpopulations in the prefrontal cortex and hippocampus [108]. Notably, this interaction is bidirectional, with microglia expressing pro-inflammatory cytokines, such as TNF-α, IL-1β, and IL-6, as well as trophic factors that mediate the interactions between the host microbiome and the brain [109].

In the setting of alcohol exposure, microglia, the resident immune cells of the CNS, play a pivotal role in neuroimmune activation and neuroinflammation [110]. Microglial activation, as evidenced by increased expression of ionized calcium-binding adaptor molecule 1 (IBA1), is a hallmark of both acute and chronic ethanol exposure in murine models, as well as in postmortem brain tissue from individuals with AUD [111,112]. This activation is mediated by a complex interplay of immune signaling pathways, including the activation of NF-κB, NADPH oxidase, and the production of reactive oxygen species [112]. Microglia are also activated by cytokine and TLR signaling, triggered by the recognition of pathogen-associated molecular patterns (PAMPs), and this is further exacerbated by the increased gut permeability and translocation of systemic inflammatory mediators caused by alcohol exposure.

Neuroinflammation is associated with mood and behavioral changes, such as the development of depression [113]. Postmortem brain tissue analyses have shown that individuals with depression exhibit increased microglial activation in cortical regions that are commonly implicated in depression [114]. Further, the administration of the IL-1β receptor antagonist IL-1Ra could mitigate anhedonic stress-induced behavior in mice [115]. While the exact neuronal mechanisms underlying this phenomenon are not yet fully understood, evidence suggests that neuroinflammation may plausibly contribute to symptoms of alcohol withdrawal and alcohol craving [116].

## 5. Gut Microbial Therapies for AUD

These recent advances in the understanding the gut–brain axis have led to several human clinical trials investigating the therapeutic use of gut microbiota-targeting interventions for the treatment of AUD and other mental health conditions that are often associated with increased alcohol consumption, with varying benefits (Table 1 and Table 2, ordered chronologically).

In the realm of prebiotics, one published trial using the prebiotic inulin in patients with severe AUD did not demonstrate any effect on alcohol craving [117]. Another study investigating the use of short chain fatty acid prebiotics in people living with HIV with and without AUD on intestinal barrier function, systemic inflammation, and brain pathology is currently recruiting (https://clinicaltrials.gov/study/NCT06139224, accessed on 16 November 2024). Colonic delivery of SCFAs also appeared to improve responses to stressors in healthy individuals [118,119]. Supplementation with probiotics also appeared to reduced symptoms of stress, anxiety, and psychological distress in stressed adults and improve depressive symptoms in patients with major depression disorder [120,121,122].

Therapies that directly modify gut microbiota include one study which used the probiotic *Lactobacillus rhamnosus* GG in patients with AUD and moderate alcohol-associated hepatitis, which showed improvements in alcohol craving [123], and another study using the probiotic VSL#3 in patients with AUD and alcohol-associated liver disease (https://clinicaltrials.gov/study/NCT05007470, accessed on 16 November 2024), which is still ongoing. Two studies using fecal microbiota transplantation (FMT) in patients with alcohol use disorder and cirrhosis also demonstrated improvements in alcohol craving [124,125]. Indirect modification of the gut microbiota using the antibiotic minocycline did not demonstrate any effect on alcohol craving in patients with AUD in one study [126], and another study examining the effects of minocycline on neuroinflammation, alcohol cue reactivity, neurocognitive performance, and alcohol use has been completed but not yet published (https://clinicaltrials.gov/study/NCT04210713, accessed on 16 November 2024). Larger and more comprehensive studies are warranted to determine the optimal delivery methods, treatment duration, and specific gut microbial targets for the effective treatment of AUD and associated mental health disorders.

Another important area of research is the development of biomarkers that can be used to (1) identify patients with AUD who are more likely to have a positive response to specific microbiome-based therapies, (2) monitoring changes in the composition and function of the gut microbiome in response to therapy, and (3) predict those who are more likely to have severe or life-threatening outcomes, such as severe alcohol withdrawal or alcohol-associated liver disease. Additionally, better understanding the impact of existing approved medications for AUD (naltrexone, acamprosate, and disulfiram in the United States, and additionally nalmefene in Europe) on the gut microbiome could provide valuable insights into the variability of treatment responses in patients with AUD.

**Table 1:** Published Human Clinical Trials Targeting the Gut Microbiome for the Treatment of AUD.

Reference	Study Design	Outcomes	Results	Limitations
Petrakis, et al., 2019 [126]	Randomized, double-blind, placebo-controlled study of 49 heavy drinkers (≥7/14 standard alcoholic drinks per week for females/males) who received placebo (n = 20), 100 mg (n = 12), or 200 mg (n = 17) of minocycline daily for 10 days	Response to alcohol Craving for alcohol Motor dexterity Cognitive function Serum cytokines	There was no effect of either dose of minocycline on alcohol craving. Minocycline treatment did not alter serum cytokine levels at baseline or during ethanol-exposure.	Effects of minocycline on quantity of alcohol consumed was not reported.
Bajaj, et al., 2021 [124]	Randomized, double-blind clinical trial of 20 patients with AUD and alcohol-associated cirrhosis who received one placebo (n = 10) or FMT (n = 10) enema from from a donor enriched in *Lachnospiraceae* and *Ruminococcaceae*	Cognition Alcohol craving Serum cytokines Fecal microbiota composition Urinary ethyl glucuronide Quality of life	In FMT group, microbial diversity and relative abundance of *Ruminococcaceae* and other SCFA-producing taxa were increased. Alcohol craving was reduced significantly in 90% of patients who received FMT, versus 30% who received placebo, at day 15.	Smaller sample size Only short-term assessment of biospecimens (15 days after intervention) No biomarker quantification of alcohol consumption at 6 months
Amadieu, et al., 2022 [117]	Randomized, double-blind, placebo-controlled study of 43 patients with severe AUD (DSM-5 ≥ 6 criteria) who received daily inulin (uptitration to 16 g per day, n = 22) or placebo (maltodextrin, n = 21) daily for 17 days	Fecal microbiota composition Serum biomarkers Evaluation of anxiety, depression, and alcohol craving Gastrointestinal tolerance questionnaire	Decreased in α-diversity with inulin Increase in relative abundance of Actinobacteria and *Bifidobacteriaceae* and decrease in *Bacteroidaceae* with inulin Increase in serum brain-derived neurotrophic factor No difference in depression, anxiety or alcohol craving in inulin group compared with placebo	32% of patients receiving placebo and 48% of patients receiving inulin experienced relapse of alcohol use during the study
Philips, et al., 2022 [125]	Retrospective analysis of 61 patients with severe alcohol-associated hepatitis who underwent FMT (n = 35) or standard of care (n = 26)	Incidence of ascites, hepatic encephalopathy, infections, and major hospitalizations	FMT was associated with increased *Bifidobacterium* and decreased *Acinetobacter*. Incidence of decompensation, infections, and major hospitalizations were higher in the standard of care group. Alcohol relapse was lower and time to relapse and 3-year survival was higher in the FMT group.	Retrospective, single-center design Confounding factors such as antimicrobial exposure were not accounted for
Vatsalya, et al., 2023 [123]	Randomized, double-blind, placebo-controlled study of 46 patients with AUD and moderate alcohol-associated hepatitis (MELD between 12 and 20) who received daily oral *Lactobacillus rhamnosus* GG (n = 24) or placebo (n = 22) for 6 months	MELD ALT AST Maddrey Discriminant Function Drinking history collected using Timeline follow-back	Treatment with *Lactobacillus rhamnosus* GG was associated with significant reduction in MELD at 1 month, as well as reduction in heavy drinking at 6 months.	Only 29 out of 46 patients completed full 6 months of medical management No biomarker quantification of alcohol consumption

ALT, alanine aminotransferase; AST, aspartate aminotransferase; DSM, Diagnostic and Statistical Manual of Mental Disorders; FMT, fecal microbiota transplantation; MELD, Model for End-Stage Liver Disease.

**Table 2:** Published Human Clinical Trials Targeting the Gut Microbiome for the Treatment of Mental Health Conditions Associated with AUD.

Reference	Study Design	Results
Chong, 2019 [120]	Randomized, double-blind, placebo-controlled study of 111 stressed adults (based on moderate stress levels using the PSS-10 questionnaire) who received either *Lactobacillus plantarum* DR7 (10^9^ CFU/day, n = 56) or placebo (n = 55) daily for 12 weeks	DR7 supplementation reduced symptoms of stress, anxiety, and psychological distress in stressed adults. DR7 reduced plasma cortisol and pro-inflammatory cytokines and increased anti-inflammatory cytokines. DR7 administration was associated with lowered expression of plasma dopamine β-hydroxylase, tyrosine hydroxylase, indoleamine 2,3-dioxygenase, and tryptophan 2,3-dioxygenase and increased expression of tryptophan hydroxylase-2 and 5-hydroxytryptamine receptor-6.
Rudzki, 2019 [121]	Randomized, double-blind, placebo-controlled study of 60 patients with major depression disorder who received either SSRIs with the probiotic *Lactobacillus Plantarum* 299v (n = 30) or SSRIs with placebo (n = 30) for 8 weeks	The LP299v group showed significant improvements in attention and memory compared to the placebo group after 8 weeks. LP299v supplementation reduced kynurenine concentrations and increased the 3-hydroxykynurenine-to-kynurenine ratio. There were no significant changes in inflammatory markers in either group.
Dalile, 2020 [118]	Randomized, triple-blind, placebo-controlled study of 65 healthy males who received colonic SCFA mixture containing 10 g (n = 22) or 20 g (n = 21) of arabinoxylan oligosaccharides or placebo daily (n = 22) for one week	Both doses of SCFAs increased serum SCFA levels, and this was associated with attenuation of the cortisol response to psychosocial stress
Tian, 2022 [122]	Randomized, double-blind, placebo-controlled study of 45 patients with major depression disorder who received *Bifidobacterium breve* CCFM1025 (freeze-dried, 10^10^ CFU of viable bacteria, n = 20) or placebo (maltodextrin, n = 25) daily for four weeks	CCFM1025 treatment exerted a superior antidepressant-like effect compared with placebo. CCFM1025 treatment was associated with significantly reduced serum serotonin turnover, compared with placebo.
Dalile, 2024 [119]	Randomized, triple-blind, placebo-controlled study of 71 healthy males who received colon-delivery capsules of 5.28 g of butyrate (n = 35) or placebo (n = 36) daily for one week	Butyrate administration increased serum butyrate levels without affecting other serum SCFAs or fecal SCFAs. Butyrate administration had a significant impact on fear memory at the subjective level, but not at the physiological level. Butyrate administration did not alter subjective or neuroendocrine responses to acute stress.

CFU, colony forming unit; SCFA, short chain fatty acid; SSRI, selective serotonin reuptake inhibitor.

## 6. Limitations to Existing Research on the Gut–Brain Axis

Despite advances in the understanding of the gut–brain axis in AUD, there are several limitations to the gut microbiome studies carried out in both preclinical models and human studies. Rodent models are hindered by their inability to recapitulate some human-specific social, environmental, and subjective factors, and more human-specific symptoms, such as alcohol craving [127]. Additionally, there are many important differences between rodent and human gastrointestinal tract anatomy and gut microbial composition that may affect the translatability of results in rodent models. For example, the murine stomach features a non-glandular forestomach, absent in humans, which stores food and harbors a high abundance of *Lactobacillus* species due to its relatively higher pH (pH 3–4) compared to the human stomach (pH 1) [128]. This, along with other anatomical differences, contributes to distinct gut microbial compositions between humans and mice. For instance, healthy humans harbor a higher abundance of the beneficial *Faecalibacterium* species, which are often reduced in individuals with excessive alcohol consumption, while *Faecalibacterium* are very rarely found in laboratory mice [129]. Notably, despite these differences, human and murine gut microbiota do share about 90% similarity in phyla, predominantly comprising Bacteroidetes and Firmicutes [130]. Nevertheless, when applying results from rodent models to human disease, these anatomic and gut microbial differences should be considered. Gnotobiotic mice offer a means to approximate the human microbiome in a murine model, but they do not perfectly replicate the conditions of the human gut [131]. Germ-free mice are raised in sterile environments and are immunocompromised, even after colonization with human microbiota [132]. Furthermore, only a subset of the human microbiota inoculum (approximately 50%) is retained in the recipient mouse microbiota, with certain genera such as *Faecalibacterium* and *Bifidobacterium* being significantly reduced or lost, while others, like *Bacteroides*, exhibit a marked increase in relative abundance following inoculation [133]. Notwithstanding these limitations, the majority of metabolomic features present in donor samples are successfully recapitulated in colonized gnotobiotic mice, suggesting that the functional properties of the gut microbiota can be effectively replicated in this model [134]. And indeed, germ-free mice colonized with microbiota from humans with cirrhosis and AUD who showed improved clinical outcomes following FMT exhibited reduced alcohol consumption and preference, suggesting that AUD-related microbiome alterations are transmissible to a gnotobiotic mouse model and can be utilized to investigate the therapeutic effects of microbiome modulation [135].

Human studies of the gut–brain axis in AUD are also subject to limitations. Most studies rely on 16S rRNA gene sequencing to compare gut microbial composition between patients with AUD and healthy controls. While this approach provides a snapshot of microbial diversity, it lacks functional insights [136,137]. To gain a deeper understanding of the gut–brain axis, future studies should employ a more comprehensive range of techniques, including metagenomic, meta-transcriptomic, and metabolomic analyses, to assess both gut microbial composition and function. Another limitation of current research is its reliance on stool samples, which neglects the distinct bacterial populations residing in the small intestine and mucosal communities [138]. Furthermore, patients with AUD often exhibit altered dietary habits and are at increased risk of malnutrition [139], which may be a confounding variable in gut microbiome analyses. For example, a study of 50 patients with AUD found that they had lower intake of essential nutrients and dietary fibers and consumed more ultra-processed foods compared to healthy subjects, which was associated with higher anxiety, lower sociability, and intestinal discomfort [140]. Chronic alcohol consumption can also injure the small intestinal mucosa and damage the brush border membrane of enterocytes where nutrients are absorbed, leading to disruption of macronutrient and micronutrient absorption [141]. Therefore, it is essential to accurately assess dietary and nutritional status in patients with AUD and consider it as a potential confounding factor in future studies.

To enhance the validity and reliability of future findings, several key concerns should be addressed. Variability in results can stem from factors such as sample size, sample timing and methodology, DNA extraction, sequencing techniques, and data analysis [142]. Moreover, studies should strive to account for confounding variables, including age, gender, antibiotic or probiotic use, diet, and supplements, to the best of their ability. Careful selection of control groups is also crucial to avoid introducing bias. By addressing these limitations, future studies can provide more accurate and reliable insights into the gut–brain axis in AUD.

## 7. Conclusions

The gut–brain axis plays a critical role in the pathophysiology of AUD, with alcohol-induced gut dysbiosis profoundly affecting microbial composition, metabolite production, and neuroimmune pathways. These disruptions contribute to neuroinflammation and addiction cycles, underscoring the gut as a promising therapeutic target. Emerging interventions, including probiotics, prebiotics, and fecal microbiota transplantation, have shown potential in reducing alcohol cravings and improving neuropsychiatric symptoms. While promising, these therapies require larger clinical trials to validate their efficacy and determine optimal application strategies. Identifying microbial biomarkers may pave the way for personalized treatments, enabling tailored interventions and better patient outcomes. However, significant knowledge gaps persist, particularly in the mechanistic understanding of gut–brain interactions in AUD. Addressing these gaps through targeted research could clarify the bidirectional impacts of alcohol on gut and brain health. Such advancements will be vital to improving therapeutic strategies and transforming our approach to this complex disorder.
